# Activated Nanocellulose from Corn Husk: Application to As and Pb Adsorption Kinetics in Batch Wastewater

**DOI:** 10.3390/polym16243515

**Published:** 2024-12-18

**Authors:** Aydeé M. Solano-Reynoso, Ruth Fany Quispe-Quispe, Yudith Choque-Quispe, Fredy Taipe-Pardo, Yovana Flores-Ccorisapra, Celia R. Yauris-Silvera, Diego E. Peralta-Guevara, Yakov Felipe Carhuarupay-Molleda, Liliana Rodriguez-Cardenas, David Choque-Quispe, Carlos A. Ligarda-Samanez

**Affiliations:** 1 Basic Sciences Department, Universidad Nacional José María Arguedas, Andahuaylas 03701, Peru; ycarhuarupay@unajma.edu.pe; 2 Research Group for the Development of Advanced Materials for Water and Food Treatment, Universidad Nacional José María Arguedas, Andahuaylas 03701, Peru; ychoque@unajma.edu.pe (Y.C.-Q.); deperalta@unajma.edu.pe (D.E.P.-G.); brysaalarcon@gmail.com (L.R.-C.); dchoque@unajma.edu.pe (D.C.-Q.); caligarda@unajma.edu.pe (C.A.L.-S.); 3 Food Nanotechnology Research Laboratory, Universidad Nacional José María Arguedas, Andahuaylas 03701, Peru; 4 Environmental Engineering Department, Universidad Nacional José María Arguedas, Andahuaylas 03701, Peru; 5 Agroindustrial Engineering Department, Universidad Nacional José María Arguedas, Andahuaylas 03701, Peru; ftaipe@unajma.edu.pe (F.T.-P.); cyauris@unajma.edu.pe (C.R.Y.-S.); 6 Systems Engineering Department, Universidad Nacional José María Arguedas, Andahuaylas 03701, Peru; yflores@unajma.edu.pe; 7 Advanced Materials Research Laboratory for Water and Food Treatment, Universidad Nacional José María Arguedas, Andahuaylas 03701, Peru; 8 Nutraceuticals and Biopolymers Research Group, Universidad Nacional José María Arguedas, Andahuaylas 03701, Peru

**Keywords:** adsorption kinetics, cellulose nanocrystals, corn husk, metal removal

## Abstract

The aim of this study was to evaluate the removal of Pb and As from an aqueous solution using corn residue cellulose nanocrystals (NCCs). The corn husk was subjected to alkaline digestion, followed by bleaching and esterification with 3% citric acid to obtain NCCs. A 10 ppm multimetal solution of Pb and As was prepared. The adsorption process was evaluated by adjusting the pH and NCC dosage, optimized through the nonlinear regression of empirical mathematical models. Based on the optimal parameters, the kinetics were evaluated using the PFO and PSO models. The NCCs displayed nanometer-level characteristics with a particle size less than 383.7 nm, a ζ potential in the range of −28–70 mV, pH_ZCP_ with an acidic tendency, a porous crystal structure as evaluated through SEM images, and the presence of functional groups with a high chelating capacity, as identified via FTIR. Optimum values of pH 8.0 and 20 mg/L of the NCC dose were found, from which it was observed that the PFO, PSO, and Elovich kinetics showed *R*^2^ > 0.974, with an adsorption capacity in the order Pb > As. The adsorbent-formulated NCCs presented a good capacity to remove heavy metals from aqueous media.

## 1. Introduction

One of the environmental problems worldwide is the contamination of water sources by heavy metals, due to the discharges of contaminated effluents that mainly come from different anthropogenic activities, such as industry, agriculture, and mining [1,2]. It has been observed that the water contamination rate can be around 200 million m^3^ per day; this fact leads to a large number of health problems that are related to heavy metals, which present high toxicity even at low concentrations, since they are persistent, bioaccumulative, and non-biodegradable, and they constitute a threat to human life, animals, and the environment [3,4,5,6].

Highly toxic metallic elements that are capable of contaminating water sources include arsenic, chromium, cobalt, nickel, copper, zinc, silver, cadmium, mercury, titanium, selenium, and lead [6,7,8]. In particular, As is a toxic and carcinogenic element at a high dose of 0.10 mg/L [9,10]. Similarly, Pb is highly toxic and non-biodegradable, and even at low concentrations, it can harm living organisms [11,12]. The presence of heavy metals in water resources causes various pathologies, poisoning, and irreparable damage to health, including muscular dystrophies, kidney stones, bone diseases, osteoporosis, Alzheimer’s disease, respiratory disorders, and carcinogenic effects [13,14].

Different methods have been developed for water treatment, including chemical, physical, and combined treatments, such as reverse osmosis, photocatalysis, electrolytic treatment, electrocoagulation, electrodialysis, electrochemical treatment, membrane technologies, and adsorption with activated carbons [15,16]. These processes generally use synthetic and inorganic inputs, which generate non-biodegradable and toxic waste, increase treatment costs, and require more specialized technologies [17,18,19].

There is an increasing demand for environmentally friendly and low-cost technologies, which also involve the use of biodegradable materials from plant waste consisting mainly of cellulose (peels, husk, leaves, and fibers) and animal waste consisting of keratin and hydroxyapatite (shells, bones, hairs, and horns); these biodegradable materials are called bioadsorbents [20,21,22,23,24].

Many residues from traditional cultivars are being used as a contribution to the circular economy [25,26]. These residues constitute raw materials with potential due to their components with specific properties. One of the alternatives is the use of a natural biopolymer, corn husk (*Zea mays* L.), due to its availability and accessibility; corn husk also has a high cellulose content, whose properties can be improved and optimized during the adsorption process when modified and activated with organic and inorganic acids to obtain cellulose nanocrystals or nanofibers, where the conformation of its functional groups would result in good efficiency in the elimination of heavy metals [27,28,29,30].

Alternatively, various materials have been developed from agricultural residues, such as orange peel, banana peel, sugar cane bagasse, rice husk, wheat straw, groundnut shell, bamboo, coffee husk, pomelo peel, pear peel, oil palm biomass waste, and fruit seeds, which are chemically modified [3,29,31,32,33,34]. These materials offer advantages due to their porous structure, high cellulose content, and the presence of functional groups that can interact with heavy metals. However, during their activation, they are subjected to treatments with strong acids (sulfuric, hydrofluoric, hydrochloric, or nitric acid), strong alkalis (sodium or potassium hydroxide), salts (ZnCl_2_), and organic compounds (polyacrylamide, polyacrylic acid, ethylenediamine, and diethylenetriamine), as well as the incorporation of nanoparticles (iron, silver, and zirconium) and surfactants [3,22,29,34,35], whose residues after being activated generate negative environmental impacts.

Cellulose modifications have been carried out through the application of strong acids, strong bases, or the combination of both, which allows for the elimination of hemicellulose and lignin [3,30,36]. However, the use of these strong substances generates waste, with a negative impact on the environment. An alternative is the use of weak acids, such as phosphoric acid, citric acid, oxalic acid, and acetic acid, which, in combination with other methods such as ultrasound, microwaves, or plasma [30,37,38,39], could improve the chelating ability of the modified cellulose [40,41].

Therefore, the research aims were to study the removal capacity of Pb and As from aqueous solution using cellulose nanocrystals and to propose and characterize a new activated material from corn husk (*Zea mays* L.), which can contribute to the environment and the circular economy.

## 2. Materials and Methods

### 2.1. Obtaining Cellulose Nanocrystals (NCCs)

NCC was provided by the research laboratory in Materials for Water and Food Treatment (LIMTA) of UNAJMA, Andahuaylas, Peru. It was prepared from corn husk, by basic digestion, in microwaves (SCP Science, Baie-d’Urfé, QC, Canada) at 140 °C with 2% sodium hydroxide (Scharlau, Barcelona, Spain) to eliminate lignin. The hemicellulose was then removed using 0.525% sodium hypochlorite (Merck, Darmstadt, Germany). Then, it was esterified in microwaves (SCP Science, Canada) at 160 °C with 4% citric acid (Scharlau, Spain) for 20 min. It was left to cool and taken to a sonicator for 15 min at 750 J and 40% amplitude (Model VCX 750, Sonics & Materials Inc., Newtown, CT, USA). Then, it was washed with abundant distilled water until reaching neutral pH. The samples were dried at 60 °C for 24 h and ground to 45 µm mesh [30].

### 2.2. NCC Characterization

Next, 50 mL of solution was prepared with pH between 3 and 12, and 0.05 mg of NCC was added, immediately agitated at 60 rpm for 24 h. At the end of the time, the pH of each solution was measured, and the data obtained were plotted as a function of the initial pH. The intersection of the curves indicates the zero charge point (ZCP) [42].

The NCC was poured into distilled water and sonicated for 1 min (Model VCX 750, Sonics & Materials Inc., Newtown, CT, USA). The sample was then placed in a polystyrene cell and transferred to a dynamic light-scattering particle size analyzer, a laser wavelength of 632.8 nm, a dispersion angle of −14.14°, and an electric field strength of 5 V/cm (ZLS-Z3000, Nicomp, Entegris, Billerica, MA, USA). Similarly, an aliquot was taken into a folded capillary cell to determine the ζ potential.

To analyze the interaction of Pb and As with the functional groups of the NCC before and after the adsorption process, tablets were prepared with 200 mg of KBr and 2 mg of NCC, then taken to an FTIR (Nicolet IS50, Thermo Fisher, Waltham, MA, USA). The readings were taken in the transmission module, at 32 scans, with an 8 cm^−1^ step in the range of 4000 at 400 cm^−1^ [43].

XRD analysis allows for obtaining structural information and crystallinity of the NCC. It was carried out using an X-ray diffractometer (model D8-Focus, Bruker, Karlsruhe, Germany) under conditions: monochromatic copper radiation (Cu Kα, λ = 1.54 Å), 2θ range from 15 to 70°, scan step 0.02°, 40 kV and 40 mA. The degree of crystallinity was calculated considering the crystalline phase’s area and the curve’s total area [44,45]. The morphology of the NCC was visualized through an SEM (Thermo Fisher, Prism E, MA, USA). The samples were placed on carbon disks under low vacuum, acceleration voltage of 30 Kv, and 3000× magnification increase

### 2.3. Evaluation of the Effect of pH and NCC Dose

From a 1000 ppm Pb and As standard solution (Scharlau, Barcelona, Spain), a 10 ppm Pb and As dilution with deionized water was prepared as a mixture, and 100 mL of the mixture was taken and adjusted with 0.1 M NaOH to pH 5.5 and 8.0. NCC (20 and 50 mg) was added while stirring at 100 rpm for 90 min. Ultimately, the remaining solution was taken to an ICPE-OES-9820 (Shimadzu, Kyoto, Japan). Pb and As readings were taken at wavelengths of 220.353 and 228.812 nm, respectively, in axial mode in triplicate, for a calibration curve with *R*^2^ > 0.998. The removal percentage (*%R*) and the adsorption capacity (*q_t_*) were determined [45,46].

(1) $$\%R=\frac{{(C}_{0}-C_{f})}{C_{0}}100$$

(2) $$q_{t}=\frac{\left(C_{0}-C_{f}\right)V}{m(g)}$$

where *%R* represents the removal percentage; *C*_0_ (mg/L) is the initial concentration of Pb and As; *C_f_* (mg/L) is the final concentration of Pb and As; *q_t_* (mg/g) is the amount of adsorbed cations; *m*(g) is adsorbent dose; *V*(L) is the metal solution volume.

### 2.4. Adsorption Process Optimization

The experimental data of the removal percentage (*%R*) were fitted to linear, quadratic, and interaction empirical models according to Equation (3).

(3) $$Y=\beta_{0}+\sum_{i=1}^{k}\beta_{i}x_{i}+\sum_{1=1}^{k-1}\sum_{j=2}^{k}\beta_{ij}x_{i}x_{j}+e_{i}$$

where *x_i_* and *x_j_* represent the independent variables (pH, NCC dose); *β_0_*, *β_i_*, and *β_ij_* are the intercept, linear, quadratic, and intercept coefficients, respectively; and *k* is the number of variables.

The fit of the models was evaluated through the fit coefficient *R*^2^. Values greater than 0.7 indicate a good fit [47,48].

### 2.5. Adsorption Kinetics Evaluation

For the adsorption kinetics tests, the optimal adsorption parameters were considered. The metal solution was adjusted to pH 8, and 20 ppm of NCC was added to 100 mL of 10 ppm metal solution, for contact times of 0, 15, 30, 60, 90, 120, 150, 200, 250, and 300 min; experiments were performed in triplicate. The samples for each time were extracted and filtered at 0.45 μm. To analyze the concentration of heavy metals, the reading was carried out in an ICP-OES [49,50].

The experimental data were fitted to kinetic models:

Pseudo first order (Equation (4)) allows us to describe the adsorption of a liquid system as a function of the capacity of a solid. Adsorption is based on the difference between the adsorption capacity at equilibrium and that obtained at a given time *t* [51,52].

Pseudo second order (Equation (5)) mainly describes the chemical interaction between adsorbent and adsorbate. It also considers that adsorption depends on the concentration of active sites [51,53,54].

The Elovich model (Equation (6)) applies to chemisorption processes and defines that the active sites of the adsorbent material are heterogeneous, which generates different activation energies, based on a second-order reaction mechanism for a heterogeneous reaction process [55,56].

The intraparticle diffusion (ID) model (Equation (7)) indicates that several steps, including diffusion within the adsorbent, can control adsorption. The sorbate diffuses from a zone of high concentration towards the surface and within the adsorbate towards the active sites (intraparticle diffusion) [57].

(4) $$q_{t}=q_{e}(1-e^{-k_{1}t})$$

(5) $$q_{t}=\frac{\left({q_{e}}^{2}k_{2}t\right)}{\left(1+q_{e}k_{2\;}t\right)}$$

(6) $$q_{t}=\frac{1}{\beta }\ln\left(1+\alpha \beta t\right)$$

(7) $$q_{t}=k_{i}t^{1/2}+C$$

where $q_{t}\;\left(mmol/g\right)$ is the adsorption capacity at time t; $q_{e}\;\left(mmol/g\right)$ is the adsorption capacity at equilibrium; $k_{1\;}({min}^{-1})$ and $k_{2\;}(g/mmol\cdot min)$ are the pseudo-first-order and pseudo-second-order rate constant, respectively; *α* (mmol/g·min) is initial adsorption rate; *β* (g/mmol) is the parameter related to the surface covered and the activation energy by chemisorption, which can take a value between 0 and 1; t (min) is the contact time; *k_i_* is the intraparticle diffusion rate (mmol/g·min^1/2^); *C* is a constant related to the average thickness of the boundary layer (mmol/g).

When *C* = 0, surface adsorption is negligible, and intraparticle diffusion completely controls the adsorption process. When *C* ≠ 0, the adsorption process takes place at both the surface and intraparticle levels. High values of *C* indicate that the adsorption process is preferentially at the surface [57,58].

Kinetic models were adjusted using nonlinear regression, considering the least squares difference between the experimental data and the data reported by the model, evaluated through the Quasi-Newton (QN) method [50].

Likewise, the adjustment coefficient *R*^2^ was calculated, and values greater than 0.7 are appropriate. Likewise, the Chi-square (*X*^2^) and mean relative error (MRE) statistics were determined, which measure the error distribution of the residuals, which were calculated with Equations (8) and (9), respectively [59,60,61]. Low values are recommended. Another criterion for evaluating kinetic models was the distribution of residuals; greater randomness is an indicator of lower systematic errors and, therefore, better fit [50].

(8) $$MRE=\frac{100}{N}{\sum_{i=1}^{N}\left|\frac{q_{adj}-q_{exp}}{q_{exp}}\right|}_{i}$$

(9) $$X^{2}={\sum_{i=1}^{N}\left[\frac{{\left(q_{exp}-q_{adj}\right)}^{2}}{q_{adj}}\right]}_{i}$$

where $q_{adj}$ is the data computed by the model; $q_{exp}$ is experimental data; *N* is data number.

### 2.6. Statistical Analysis

The data were analyzed through an ANOVA and principal component analysis, at a 5% significance level. The comparison of the mean of treatments was also evaluated through the Tukey test. Statistica V12 software, Solver add-in, and Excel spreadsheets were used.

## 3. Results and Discussions

### 3.1. Zero Charge Point (ZCP) and ζ Potential

**Figure 1:**
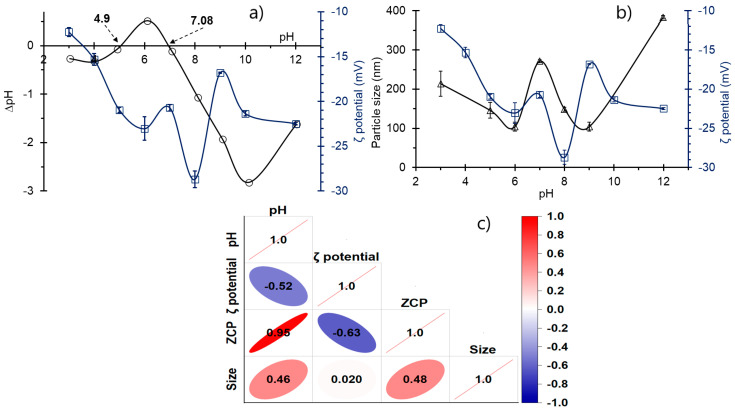
(**a**) Zero charge point and ζ potential of NCC, (**b**) particle size and ζ potential of NCC, (**c**) pH-ZCP-ζ potential-size correlation.

The ZCP identifies the pH value at which the NCC can adsorb metal cations. As shown in Figure 1a, two intersection points were observed, 4.9 (pH_ZCP1_) and 7.08 (pH_ZCP1_). These values suggest the presence of two charge-neutral or isoelectric states on the adsorbent, indicating a heterogeneous surface with regions of differing chemical properties. This amphoteric behavior enables the material to function in both acidic and basic media, allowing it to adsorb cationic and anionic contaminants [62,63,64]. The pH_ZCP1_ (4.9) is likely associated with the esterified portion of cellulose, derived from citric acid, which has dissociation constants of pKa_1_ 3.13 and pKa_2_ 4.76. In contrast, pH_ZCP2_ 7.08 may be attributed to the residual hydroxyl groups resulting from sodium hydroxide treatment. These groups are neutralized by the acidic component of citric acid, forming a salt, further enhancing the material’s adsorption properties.

When the pH is below the pH_ZCP_, the surface charge of the adsorbent becomes positive, which hinders interaction with positively charged substances in the solution due to electrostatic repulsion. Conversely, when the pH is above the pH_ZCP_, the adsorbent surface acquires a negative charge, significantly enhancing its capacity to adsorb cationic ions [43,65,66,67].

The attachment of free carboxylic groups from citric acid to cellulose increases the negative charge of the surface of the adsorbent. Each molecule of citric acid contributes two additional carboxylic sites to the cellulose surface, thereby enhancing its binding capacity for cationic species. This behavior is characteristic of cellulosic materials, which possess negatively charged functional groups that facilitate interaction with cationic contaminants [22,68,69,70,71].

The stability of NCC in an aqueous medium was assessed using ζ potential measurements, which ranged from −28.70 to −12.26 mV (Figure 1a). These results indicate the strong anionic charge of NCC, enabling it to disperse effectively in water and enhancing its capacity to adsorb cationic metals [67,71,72,73]. While no clear trend was observed, the lowest ζ potential value was recorded at pH 8. Additionally, a strong inverse correlation (−0.63) was identified between the ZCP and ζ potential (Figure 1c), further supporting the relationship between surface charge characteristics and the adsorption efficiency of NCC. 

### 3.2. Particle Size

The size distribution of NCC ranges from 104.2 to 383.7 nm across a pH range of 3 to 12 in an aqueous medium (Figure 1b), confirming its nanometric size. This size range is attributed to the prior treatments of the cellulose, which enables the dispersion of the amorphous fraction, resulting in hydrolysis and the formation of crystalline structures. As a result, the material exhibits a larger surface area and enhanced porosity [74]. These characteristics allow for improved electrostatic interactions during the adsorption process and reduce adsorption energy [75,76]. The particle size shows a slight positive correlation with ζ potential (Figure 1b,c). This may be due to smaller particles exhibiting better stability, as they offer a greater number of functional groups with partial electrical charges and larger contact surfaces. Such a relationship has been previously observed in cellulosic materials [44,67,77,78].

### 3.3. Effect of pH and Adsorbent Dose on the Removal of As and Pb

The results showed that NCC exhibited a strong affinity for metal cations, removing up to 58.05% of As and 83.43% of Pb, with no significant difference observed between treatments for either metal (Table 1). pH and dose did not significantly influence the removal efficiency; however, the interaction effect between these factors was significant (*p*-value < 0.05) (Table 2). This phenomenon is influenced by the previous treatment of cellulose, as well as factors such as the zero charge point and ζ potential [79,80,81,82]. 

**Figure 2:**
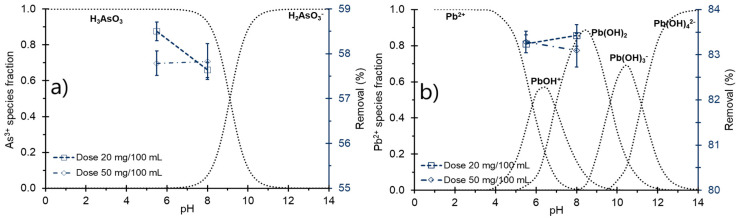
Species diagram to pH and removal percentage, (**a**) As^3+^ (with dissociation constants: log K_1_ = −9.2, log K_2_ = −12.1, log K_3_ = −13.4 [91]), (**b**) Pb^2+^ (with dissociation constants: log K_1_ = −7.7, log K_2_ = −17.1, log K_3_ = −28.1, log K_4_ = −39.7 [92]).

The acid at pH between 5 and 7 dissociates, forming an anionic species (Figure 2). This form is more readily removed, as NCC exhibits amphoteric behavior between PH_ZCP1_ and PH_ZCP2_, allowing for the formation of electrochemical bonds between the functional groups, which enhances the adsorption of As [83,84].

The adsorption of Pb showed minimal variation with increasing pH. However, at alkaline pH and lower doses of NCC, the removal efficiency increased slightly. This behavior can be explained by the fact that, above PH_ZCP2_, the NCC surface becomes more negatively charged. At this pH, the hydroxylated form of Pb (PbOH^+^) predominates (Figure 2), facilitating the formation of ion–dipole bonds between the Pb species and the NCC surface. Such behavior is characteristic of high adsorbent doses. This is primarily due to the increased availability of active sites, improved selectivity, and increased surface area [81,85,86,87,88].

At pH 8, both PbOH^+^ and Pb^2+^ forms are adsorbed primarily through electrostatic interactions and ion exchange. In contrast, the Pb(OH)_2_ form may be adsorbed via hydrogen bonding and coordination interaction with the ester groups of cellulose, which are enhanced by the chemical modification of the adsorbent [36,70,76,89,90].

One advantage of using high-porosity cellulosic materials is their ability to be easily desorbed or regenerated in an aqueous medium at low pH, allowing for reuse. However, the adsorption effectiveness may decrease over time due to the fixation of some adsorbates through high-energy chemical bonds.

Pb exhibits a higher affinity for adsorption than As due to its greater polarization, which allows it to readily form covalent bonds with the active sites of NCC. This is further enhanced by Pb’s larger ionic radius and its speciation, which facilitates the rapid formation of internal spherical complexes. These factors increase interaction with the adsorbent, providing it with both thermodynamic and kinetic advantages [45,50,93,94].

Several mechanisms contribute to the adsorption of the metal ions, including the following: (1) Electrostatic interactions: the surface charge of NCC facilitates attractions or repulsions depending on the pH, with stronger attraction observed at acidic pH for As and slightly weaker attraction for Pb. (2) Selective ion exchange: metal ions can be exchanged with functional groups of the polymer, which is influenced by both pH and ionic strength. (3) Coordination and complexation: specific functional groups on the studied polymers can form stable complexes with the metal ion, promoting physisorption through Van Der Waals forces, which aid in the retention of heavy metal. (4) Swelling effects: water uptake and the porosity of the adsorbent influence the accessibility and transport of metal ions to the adsorption sites [95,96,97,98]. These mechanisms provide insights into the factors affecting metal ion removal, enabling the establishment of operational parameters for designing efficient selective removal systems.

In real wastewater, the coexistence of dissolved cations can generate synergistic effects that enhance the fixation of Pb and As on the NCC surface by forming stable complexes, reducing the likelihood of immediate desorption (3). However, the presence of cations, such as Ca^2+^, Mg^2+^, Fe^3+^, and Cu^2+^, introduces competition for active adsorption sites, leading to reduced adsorption of Pb and As [99]. Similarly, the presence of anions like phosphate can decrease the adsorption of As by forming arsenate–phosphate complexes [100].

Moreover, organic compounds such as humic and fulvic acids can adsorb onto the NCC surface, altering its surface properties and reducing its adsorption capacity for Pb and As. These acids also affect the speciation of Pb and As in solution by modifying the pH of the medium, thereby indirectly affecting their adsorption [101,102].

### 3.4. XDR Diffractogram and Functional Group Identification

**Figure 3:**
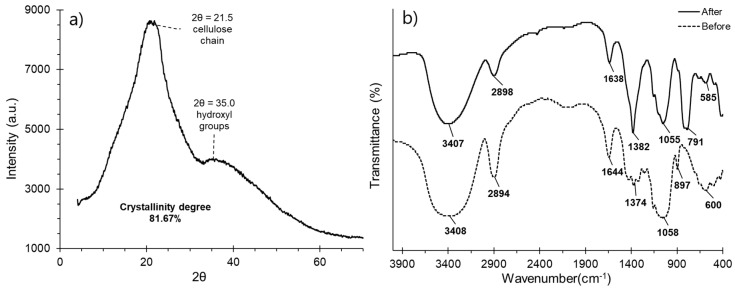
(**a**) NCC XRD diffractogram, (**b**) FTIR spectra before and after the NCC adsorption process.

X-ray diffraction analysis revealed a prominent 2θ peak at 21.5 (Figure 3a), corresponding to the cellulose structure, and another at 35.0, which indicates the presence and availability of hydroxyl groups [44,103]. The crystallinity index (CI) of the NCC was calculated to be 81.67%, reflecting a highly ordered and compact structure. This high CI is attributed to the exposure of citrate groups, which facilitate crystal formation and result in broad diffraction peaks (Figure 3a). The high crystallinity of the NCC suggests a strong potential for metal adsorption, as it enhances swelling behavior and provides a larger contact surface for interaction with metal anions [4,38,41,44].

FTIR analysis was used to identify the functional groups of NCC before and after the adsorption process. Before adsorption, a prominent peak at 3408 cm^−1^ was observed, corresponding to hydroxyls groups, which are the primary component of cellulose polymers (Figure 3b). The intensity of this peak decreased significantly after adsorption, indicating the complexation of Pb and As with OH groups [30,69,104]. Peaks at 2898, 1430, and 897 cm^−1^ correspond to the methyl and methylene groups in the activated cellulose structure. The peak at 897 cm^−1^, associated with the amorphous region of NCC, showed a marked decrease following Pb and As adsorption. Additionally, the peak at 1430 cm^−1^, representing part of the NCC crystalline arrangement, disappeared entirely after adsorption, suggesting disruption of the structure caused by the Pb and As metal ions [30,69]. The peak at 1644 cm^−1^, attributed to the stretching vibration of the ester and carbonyl bonds as well as the bending of the hydroxyl groups from adsorbed water, also decreases significantly after adsorption. This reduction indicates the displacement of hydroxyl groups by metal ions, forming stronger complexes than the hydrogen bonds, highlighting the effectiveness of the adsorption process [50,105,106]. 

Likewise, the peak at 1374 cm^−1^ indicates CH deformation, with low intensity in NCC, and after adsorption, it shifts towards 1382 cm^−1^ with high intensity, indicating changes in the chemical environment of the CH groups due to the formation of complexes coordinated with the metal ions Pb and As, modifying the conformation of the cellulose structure and the network of hydrogen bonds, confirming the high adsorption capacity, mainly for Pb [49,104]. Although the peak at 1410 cm^−1^ disappears after adsorption, this is due to the changes in the mobility of the CH_2_ groups and the structural reorganization caused by the presence of the metal. On the other hand, the peak around 1058 cm^−1^, corresponding to the C-O stretching related to the pyranose ring and linked to the C-O-C glycosidic stretching, slightly decreases in intensity after adsorption due to the direct metal–oxygen interaction, which generates a weakening of the C-O bond and the formation of metal-C-O coordinated complexes, which would modify the structure of the pyranose ring and the glycosidic bonds [107,108]. Finally, the peak below 600 cm^−1^, corresponding to the out-of-plane deformation vibrations due to OH groups, shifted towards 791 cm^−1^ after adsorption due to the energetic increase in the metal–cellulose interaction force, specifically the bond with oxygen, the same as at 585 cm^−1^ [45,50,69].

### 3.5. Morphological Analysis

**Figure 4:**
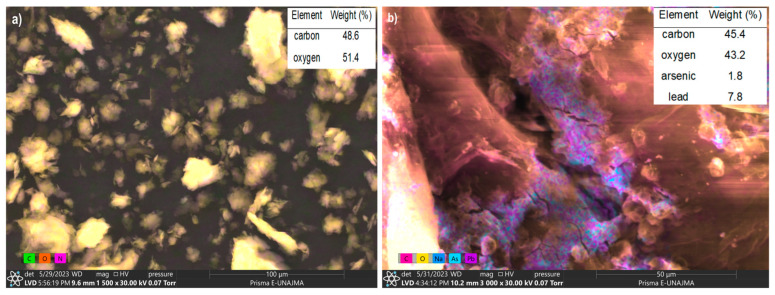
(**a**) SEM morphology of the NCC before adsorption, (**b**) SEM morphology of the NCC after adsorption.

Figure 4a illustrates the morphology of NCC before the adsorption process, with an elemental composition of carbon (48.6%) and oxygen (51.4%), which is typical for this type of material [106,109]. The structure was altered by the applied treatments, resulting in the formation of nanocrystals with sharp laminar contours, high porosity, and a solid and agglomerate structure. These characteristics are conducive to an effective metal adsorption process and are typical of cellulosic nanomaterials [30,103,106,110]

The sharp laminar shape observed in the NCC is attributed to the combined effects of high-temperature, microwave, and acid treatments. These processes cause the rupture of cellulosic fibers and the removal of non-cellulosic components. This structural modification enhances heterogeneous adsorption by increasing the availability of interface and pore accessibility within the adsorbent material [104,110,111].

Figure 4b presents the morphology after the adsorption process. Fixation of the metal ions of As (1.8%) and Pb (7.8%) was observed, forming chelating complexes, adsorbed on NCC, through electrostatic attraction forces and the active centers of the NCC. In this way, the chelating activity of the esterified cellulosic materials is confirmed [106,112,113].

### 3.6. Adsorption Process

Various models were tested, with their coefficients and *R^2^* values summarized in Table 3. The results indicate that the linear model with interaction achieved the highest value, demonstrating superior predictive accuracy. Unlike the quadratic model with interaction, the linear model’s coefficient was non-zero, highlighting the significance of the linear variables in capturing the system’s behavior. This emphasizes the utility of linear variables for understanding and simulating the adsorption process [114].

The optimal removal percentage was determined through the linear model with interaction, based on the criteria presented in Table 4. The model predicted optimal conditions at pH 8 and an NCC dose of 20 mg/100 mL, with estimated adsorption rates of 57.44% for As and 83.42% for Pb. Experimentally, these conditions yielded removals of 25.22% for As and 99.99% for Pb. The discrepancy between predicted and experimental values is attributed to intrinsic factors, such as electrostatic interactions, zeta potential, particle size, agitation of the aqueous system, as well as extrinsic factors like ambient temperature. This variation is typical for batch adsorption systems [115,116,117].

Comparatively, other studies have reported Pb removals of up to 97% and 91% for As with cellulosic materials that have been subjected to more aggressive treatments, including strong acids, strong bases, or carbonization, or that have used higher doses of adsorbent [118,119,120,121]. Despite the less intensive preparation methods used in this study, the obtained results are promising and highlight the potential of the NCC material.

The nanometric size of the obtained NCC offers several advantages in industrial applications beyond metal ion adsorption. Its ability to form nanoporous networks can enhance filtration processes, improving efficiency and performance. Additionally, the excellent suspension stability, as demonstrated by the ζ potential measurements, suggests that NCC could serve as an effective coadjutant in flocculation–coagulation processes and treatment of water with oily residues. This property also makes it suitable for treating water contaminated with oily residues, further expanding its potential applications in water treatment technologies.

The hydroxyl and carboxyl groups presented in the NCC structure allow for the exchange of hydrogen ions for Pb^2+^ and As^3+^ ions, facilitating the formation of coordinated complexes with stable bonds. However, the efficiency of these interactions is influenced by the pH of the medium. At pH values above the ZCP, electrostatic attractions become prominent, further enhancing the adsorption capacity.

The initial pH of the metal solution plays a critical role in the removal process, with its effectiveness also influenced by the adsorbent dosage. Furthermore, the activation mechanism of the adsorbents, including the availability of chelating groups and the physical characteristics of the particles, significantly impacts performance. Notably, the results obtained in this study are slightly higher than those reported in previous studies (Table 4). These variations can be attributed primarily to differences in the initial concentration of the metal ions.

**Table 4:** Optimal factor values for NCC in batch.

Cellulosic Material	Parameters	Restrictions			Experimental Application	Removal					
		Min	Max	Optimum		Predicted		Experimental			
						As (%)	Pb (%)	As (mmol/g)	As (%)	Pb (mmol/g)	Pb (%)
NCC (This work)	pH	5.5	8.0	8.0	8.00	57.44	83.42	2.18 ± 0.04	25.22 ± 0.36	2.49 ± 0.01	99.99 ± 0.01
	Dose (mg/L)	20.0	50.0	20.0	20.0						
Cellulose/Chitin beads [122]	pH	Multimetal solution			4.0					0.33	
	Dose (mg/L)				1000						
Citric acid-modified celluloses [11]	pH	Unimetal solution			5.0						94.0
	Dose (mg/L)				2500						
Tricarboxylic cellulose nanofiber [27]	pH	Multimetal solution			5.5					0.47	
	Dose (mg/L)				1000						
Alginate/Nanocellulose Beads [36]	pH	Multimetal solution			6.0					0.39	95.0
	Dose (mg/L)				1000–2000						
Poly(acrylonitrile-co-methacrylic acid)-g-cellulosic okra fibers [46]	pH	Multimetal solution			5.5					≈0.55	
	Dose (mg/L)				1000						
Sugarcane bagasse/Citric acid/Fe3O4 [70]	pH	Multimetal solution			≈7.0					0.56	≈90.0
	Dose (mg/L)				1666						
Sugarcane Bagasse/Corn Stalk Biomass [76]	pH	Unimetal solution			5.0–6.0						>98.0
	Dose (mg/L)				1000						
Iron–Manganese Oxide Incorporated Active Rice Husk Silica [119]	pH	Multimetal solution			4.0			0.13			
	Dose (mg/L)				300						
Rye husk [120]	pH	Multimetal solution			---						97.2
	Dose (mg/L)				3000						
Banana Peels [81]	pH	Unimetal solution			5.0						98.15
	Dose (mg/L)				550						
Cellulose-N(4)-Antipyrinyl Thiosemicarbazide [113]	pH	Multimetal solution			5.0						≈100.0
	Dose (mg/L)				70						

### 3.7. Adsorption Kinetics

The study of adsorption kinetics is crucial for designing efficient adsorption systems and enables the determination of residence times, reaction rates, and reactor dimensions [110,123]. The optimal removal parameters were applied to develop the adsorption kinetics of As and Pb.

The PFO model describes the adsorption of systems in the liquid–solid phase as a function of adsorption capacity, considering that the rate of occupation of the binding site is proportional to the number of unoccupied sites on the sorbent [124]. It was observed that the model reported *R*^2^ 0.995 and 0.988 for As and Pb, respectively, low *MRE* and *X*^2^ values, and random residual dispersion; these values are indicative of a good fit. The adsorption rate *k*_1_ reported values of 0.034 and 0.068 min^−1^ for As and Pb, respectively (Table 5). Values between 0.001 and 0.1 min^−1^ are indicative of moderate to rapid adsorption [125]. Thus, NCC presents good adsorption, mainly for Pb, although this could be conditioned by factors of the adsorption system, such as pH, nature of the adsorbent, concentration of metal ions, and temperature [125,126].

The equilibrium adsorption capacity (*q_e_*), representing the maximum amount of moles adsorbed by the NCC, was found to be 6.210 and 2.453 mmol/g of As and Pb, respectively. These values are characteristics of porous materials with high surface areas and nanometric dimensions [127,128]. This suggests that the adsorption process is primarily governed by chemisorption, involving the formation of chemical bonds between the functional groups of the NCC and the metal ions, which requires high activation energy. However, physisorption interactions between the adsorbate and the adsorbent also contribute to the overall adsorption process [129,130,131,132]. Notably, the values of *q_e_* found for multimetal adsorption systems were higher than those reported for pretreated and activated cellulosic materials [127,128,133].

On the other hand, the PSO model allows for describing the adsorption capacity in the solid phase and the chemical interaction between the adsorbent and adsorbate, because the individual molecules adhere to the adsorption sites on the surface [55,134]. This model reported *R*^2^ values close to unity for both metal ions, with low *MRE* and *X*^2^ values (Table 5); this behavior is common for multimetal adsorption systems. [106,112,135].

**Figure 5:**
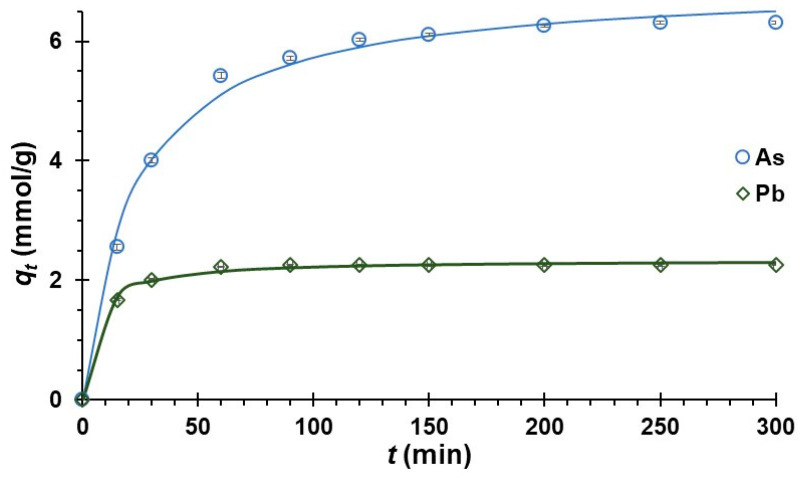
Kinetic curves for Pb and As.

The affinity of the adsorbent for the adsorbate was assessed using the constant *k_2_* [126,127], which reported values of 0.007 for As and 0.047 g/mmol.min for Pb. The higher value of *k_2_* suggests a faster adsorption rate for this metal. The rapid stage of adsorption was observed within the first 15 min (Figure 5). The equilibrium adsorption capacity (*q_e_)* reached 6.984 and 2.600 mmol/g for As and Pb, respectively. These values, which surpass those reported for other cellulosic materials, indicate that the NCC possesses adequate surface area and porosity, enabling better accessibility to the functional groups of the adsorbent [41,128,132,136,137]. These values are typical for treated and activated materials of vegetable origin [128,132]. The results suggest that sorption processes are completely governed by chemical reactions, including phenomena such as electron exchange and covalent forces [131,132,138].

The Elovich model applies to multimetallic systems with adsorbents with heterogeneous surfaces. *R*^2^ values > 0.974 were reported, indicating a good fit. The parameter *α*, which is related to the initial adsorption rate, was 1.027 and 18.058 g/mmol·min for As and Pb, respectively.

Being higher for Pb suggests that NCC has a preference for this. On the other hand, *β,* which is closely related to the activation energy in the chemisorption process [139], was higher for Pb (3.790 g/mmol), which confirms the affinity for this metal.

The intraparticle diffusion model explains the transport of adsorbate molecules through the porous structure of the adsorbent, occurring in three sequential stages: (a) diffusion in the external film, (b) intraparticle diffusion, and (c) adsorption onto the internal active sites. This transport can be assessed using the *C* parameter [57,94,140], which was found to be higher for As (Table 5). Although the *C* values are below 10 mmol/g, this indicates a moderate effect on the boundary layer, suggesting that metals rapidly adsorbed on the surface while intraparticle diffusion occurred more slowly. This is further supported by the relatively low kid values observed [58,141]. Although the model reported a low *R^2^*, it is nevertheless higher than 0.7.

The results obtained are promising, particularly due to the use of non-toxic activating substances and the use of ultrasound, which have minimal environmental impacts. While activation with substances, such as strong bases, strong acids, iron salts, EDTA, among others, allows for improving porosity, the surface area, availability of active sites, stability, increases in chelating groups, mechanical and chemical resistance, and affinity for certain metals translate into better ion adsorption in multimetal aqueous systems [34,64,71,113,142]. These produce wastes that negatively impact the environment and do not contribute to sustainability and the circular economy [94], so it is necessary to broaden the study horizons, based on sustainable and environmentally friendly chemical processes.

Another aspect to consider is the adsorption capacity after regeneration and reuse. The regeneration efficiency of NCC being a cellulosic material is usually high, reaching between 80 and 95% of the initial capacity after the first regeneration [127,143]. Although these experiments were not performed in the present study, the regenerative capacity of NCC could be considerable due to the activation of this material.

## 4. Conclusions

Corn husk residues subjected to microwave esterification in citric acid were used to produce cellulose nanocrystals (NCCs) with nanoscale properties. These NCCs exhibited moderate stability in an aqueous medium, zero charge points of 4.9 and 7.08, and functional groups with chelating capabilities. In adsorption tests for Pb and As in an aqueous system, optimal conditions were found at pH 8.0 and an NCC dose of 20 mg/L, achieving removal rates of 25.22% for As and 99.99% for Pb. The kinetic evaluation revealed a rapid adsorption rate during the first 15 minutes, with the data fitting well to the PFS, PSO, and Elovich models. The model parameters indicated that the adsorption process is primarily driven by chemisorption processes, with NCCs exhibiting a heterogeneous surface and a high availability of active sites. Additionally, intraparticle diffusion was found to be slow. The production of NCCs represents a promising method for creating cellulosic materials with high adsorption capacity for metal ions in aqueous environments.

## Figures and Tables

**Table 1:** As and Pb removal (%).

Treatment	pH	NCC Dose (mg/100 mL)	As^3+^					Pb^2+^				
			$\bar{x}$	±	S	CV	*	$\bar{x}$	±	S	CV	*
T1	5.5	20	58.05	±	0.21	0.36	A	83.24	±	0.20	0.24	A
T2	5.5	50	57.79	±	0.27	0.47	A,B	83.28	±	0.24	0.29	A
T3	8.0	20	57.64	±	0.17	0.30	B	83.43	±	0.24	0.28	A
T4	8.0	50	57.83	±	0.40	0.70	A,B	83.10	±	0.37	0.45	A

Where $\bar{x}$, mean; S, standard deviation; CV, variability coefficient (%), for n = 3. * Different letters indicate significant difference, evaluated by Tukey’s test at 5% significance.

**Table 2:** Effect of pH and dosage on As and Pb removal.

Source	*p*-Value		Criterion
	As	Pb	
pH	0.075	0.997	NS
Dose (ppm)	0.723	0.144	NS
pH-Dose (ppm)	0.019	0.046	S

Where: NS, not significant; S, significant.

**Table 3:** As and Pb coefficients.

Model	*β_0_*	*a*	*b*	*c*	*d*	*e*	*R^2^*
As							
Linear	58.3635	−0.0733	−0.0012	---	---	---	0.3991
linear with interaction	59.8065	−0.2871	−0.0424	0.0061	---	---	1.0000
Quadratic with interaction	58.2649	0.0000	0.0000	0.0061	−0.0213	−0.0006	1.0000
**Pb**							
Linear	83.4249	0.0001	−0.0047	---	---	---	0.3634
linear with interaction	82.2484	0.1744	0.0289	−0.0050	---	---	1.0000
Quadratic with interaction	83.2299	0.0000	0.0000	−0.0050	0.0129	0.0004	1.0000

Where: *a*, pH coefficient; *b*, NCC dose coefficient; *c*, pH and dose coefficient; *d*, pH coefficient squared; *e*, dose coefficient squared.

**Table 5:** Parameters of adsorption kinetic models.

Metal	Pseudo First Order					
	*q_e_*	*k*_1_	*R* ^2^	*MRE*	*X* ^2^	Residuals
As	6.210	0.034	0.995	2.406	0.043	Random
Pb	2.453	0.068	0.988	2.825	0.032	Random
**Pseudo second order**						
	***q_e_***	***k*** **_2_**	***R*** **^2^**	***MRE***	***X*** **^2^**	**Residuals**
As	6.984	0.007	0.998	1.961	0.028	Random
Pb	2.600	0.047	0.999	1.339	0.007	Random
**Elovich**						
	***α***	***β***	***R*** **^2^**	***MRE***	***X*** **^2^**	**Residuals**
As	1.027	0.810	0.974	5.576	0.220	Random
Pb	18.058	3.790	0.984	3.451	0.038	Slightly Random
**Intraparticle diffusion**						
	***k_i_***	***C***	***R*** **^2^**	***MRE***	***X*** **^2^**	**Residuals**
As	0.244	2.782	0.810	10.455	0.730	Tendentious
Pb	0.032	1.815	0.751	4.408	0.067	Tendentious

## Data Availability

The original contributions presented in the study are included in the article, further inquiries can be directed to the corresponding authors.

## References

[1] Choque-Quispe D., Herbas-De la Cruz R.K., Ligarda-Samanez C.A., Solano-Reynoso A.M., Buleje-Campos D., Choque-Quispe Y., Muñoz-Saenz J.C., Pumacayo-Sanchez Z.O., Sumarriva-Bustinza L.A., Ticona N.A.S. (2024). Caffeine, surfactants and organic matter in a high Andean River: Chumbao River case, Apurimac, Peru. Case Stud. Chem. Environ. Eng..

[2] Ramos-Pacheco B.S., Choque-Quispe D., Ligarda-Samanez C.A., Solano-Reynoso A.M., Choque-Quispe Y., Landa J.P.A., Cerna H.W.A., Palomino-Rincón H., Taipe-Pardo F., Zamalloa-Puma M.M. (2023). Water Pollution Indexes Proposal for a High Andean River Using Multivariate Statistics: Case of Chumbao River, Andahuaylas, Apurímac. Water.

[3] Jamshaid A., Hamid A., Muhammad N., Naseer A., Ghauri M., Iqbal J., Rafiq S., Shah N.S. (2017). Cellulose-based Materials for the Removal of Heavy Metals from Wastewater—An Overview. ChemBioEng Rev..

[4] Awang N.A., Salleh W.N.W., Yusof N., Karim Z.A., Ismail A.F. (2021). Nanocellulose-Based Materials for Heavy Metal Removal from Wastewater. Environ. Nanotechnol..

[5] Pabón S.E., Benítez R., Sarria R.A., Gallo J.A. (2020). Water contamination by heavy metals, analysis methods and removal technologies. A review. Entre Cienc. E Ing..

[6] Sable H., Singh V., Kumar V., Roy A., Pandit S., Kaur K., Rustagi S., Malik S. (2024). Toxicological and bioremediation profiling of nonessential heavy metals (mercury, chromium, cadmium, aluminium) and their impact on human health: A review. Toxicol. Anal. Clin..

[7] Bayuo J., Rwiza M., Mtei K. (2022). A comprehensive review on the decontamination of lead (II) from water and wastewater by low-cost biosorbents. RSC Adv..

[8] Vijayaraghavan K., Balasubramanian R. (2015). Is biosorption suitable for decontamination of metal-bearing wastewaters? A critical review on the state-of-the-art of biosorption processes and future directions. J. Environ. Manag..

[9] Armah F.A., Quansah R., Luginaah I. (2014). A systematic review of heavy metals of anthropogenic origin in environmental media and biota in the context of gold mining in Ghana. Int. Sch. Res. Not..

[10] Upadhyay S., Sinha A. (2021). A study on different bioremediation approaches to hexavalent chromium. Pollution Control Technologies: Current Status and Future Prospects.

[11] Kuo C.-Y., Wu C.-H., Chen M.-J. (2015). Adsorption of lead ions from aqueous solutions by citric acid-modified celluloses. Desalination Water Treat..

[12] Tunali S., Akar T., Özcan A.S., Kiran I., Özcan A. (2006). Equilibrium and kinetics of biosorption of lead (II) from aqueous solutions by Cephalosporium aphidicola. Sep. Purif. Technol..

[13] Mahurpawar M. (2015). Effects of heavy metals on human health. Int. J. Res. Granthaalayah.

[14] Duan B., Feng Q. (2021). Comparison of the potential ecological and human health risks of heavy metals from sewage sludge and livestock manure for agricultural use. Toxics.

[15] Lin S.H., Kiang C.D. (2003). Combined physical, chemical and biological treatments of wastewater containing organics from a semiconductor plant. J. Hazard. Mater..

[16] Gogate P.R., Pandit A.B. (2004). A review of imperative technologies for wastewater treatment II: Hybrid methods. Adv. Environ. Res..

[17] Noor A., Kutty S.R.M., Isa M.H., Farooqi I.H., Affam A.C., Birniwa A.H., Jagaba A.H. (2023). Treatment innovation using biological methods in combination with physical treatment methods. The Treatment of Pharmaceutical Wastewater.

[18] Oller I., Malato S., Sánchez-Pérez J. (2011). Combination of advanced oxidation processes and biological treatments for wastewater decontamination—A review. Sci. Total Environ..

[19] Norrrahim M.N.F., Kasim N.A.M., Knight V.F., Misenan M.S.M., Janudin N., Shah N.A.A., Kasim N., Yusoff W.Y.W., Noor S.A.M., Jamal S.H. (2021). Nanocellulose: A bioadsorbent for chemical contaminant remediation. RSC Adv..

[20] Mukherjee I., Singh U.K., Singh R.P. (2021). An overview on heavy metal contamination of water system and sustainable approach for remediation. Water Pollution and Management Practices.

[21] Selvasembian R., Thokchom B., Singh P., Jawad A.H., Gwenzi W. (2024). Remediation of Heavy Metals: Sustainable Technologies and Recent Advances.

[22] Malik D.S., Jain C.K., Yadav A.K. (2017). Removal of heavy metals from emerging cellulosic low-cost adsorbents: A review. Appl. Water Sci..

[23] Rojas-Mayorga C.K., Mendoza-Castillo D.I., Bonilla-Petriciolet A., Silvestre-Albero J. (2016). Tailoring the adsorption behavior of bone char for heavy metal removal from aqueous solution. Adsorpt. Sci. Technol..

[24] Sun B., Tian H.Y., Zhang C.X., An G. (2013). Preparation of biomimetic-bone materials and their application to the removal of heavy metals. AIChE J..

[25] Barampouti E.M., Mai S., Moustakas K., Malamis D., Loizidou M. (2021). Status and perspectives of agricultural residues in a circular and resource-efficient context. Clean Energy and Resources Recovery.

[26] Adil M., Nasir A., Sikandar A., Khan N.M. (2022). Recent Advances in Circular Bioeconomy. Waste-to-Energy.

[27] Abou-Zeid R.E., Dacrory S., Ali K.A., Kamel S. (2018). Novel method of preparation of tricarboxylic cellulose nanofiber for efficient removal of heavy metal ions from aqueous solution. Int. J. Biol. Macromol..

[28] Kurniawan T.W., Sulistyarti H., Rumhayati B., Sabarudin A. (2023). Cellulose Nanocrystals (CNCs) and Cellulose Nanofibers (CNFs) as Adsorbents of Heavy Metal Ions. J. Chem..

[29] Kaur J., Sengupta P., Mukhopadhyay S. (2022). Critical review of bioadsorption on modified cellulose and removal of divalent heavy metals (Cd, Pb, and Cu). Ind. Eng. Chem. Res..

[30] Choque-Quispe D., Choque-Quispe Y., Ligarda-Samanez C.A., Peralta-Guevara D.E., Solano-Reynoso A.M., Ramos-Pacheco B.S., Taipe-Pardo F., Martínez-Huamán E.L., Aguirre Landa J.P., Agreda Cerna H.W. (2022). Effect of the addition of corn husk cellulose nanocrystals in the development of a novel edible film. Nanomaterials.

[31] Sobhanardakani S., Parvizimosaed H., Olyaie E. (2013). Heavy metals removal from wastewaters using organic solid waste—Rice husk. Environ. Sci. Pollut. Res..

[32] Farooq U., Khan M.A., Athar M., Kozinski J.A. (2011). Effect of modification of environmentally friendly biosorbent wheat (*Triticum aestivum*) on the biosorptive removal of cadmium (II) ions from aqueous solution. Chem. Eng. J..

[33] Buasri A., Chaiyut N., Tapang K., Jaroensin S., Panphrom S. (2012). Equilibrium and kinetic studies of biosorption of Zn (II) ions from wastewater using modified corn cob. Apcbee Procedia.

[34] Garba Z.N., Lawan I., Zhou W., Zhang M., Wang L., Yuan Z. (2020). Microcrystalline cellulose (MCC) based materials as emerging adsorbents for the removal of dyes and heavy metals—A review. Sci. Total Environ..

[35] Rocky M.M.H., Rahman I.M.M., Biswas F.B., Rahman S., Endo M., Wong K.H., Mashio A.S., Hasegawa H. (2023). Cellulose-based materials for scavenging toxic and precious metals from water and wastewater: A review. Chem. Eng. J..

[36] Abou-Zeid R.E., Ali K.A., Gawad R., Kamal K.H., Kamel S., Khiari R. (2021). Removal of Cu (II), Pb (II), Mg (II), and Fe (II) by adsorption onto alginate/nanocellulose beads as bio-sorbent. J. Renew. Mater..

[37] Rana A.K., Frollini E., Thakur V.K. (2021). Cellulose nanocrystals: Pretreatments, preparation strategies, and surface functionalization. Int. J. Biol. Macromol..

[38] Jiang H., Wu S., Zhou J. (2023). Preparation and modification of nanocellulose and its application to heavy metal adsorption: A review. Int. J. Biol. Macromol..

[39] Raza M., Abu-Jdayil B. (2022). Cellulose nanocrystals from lignocellulosic feedstock: A review of production technology and surface chemistry modification. Cellulose.

[40] Mohamed R.R. (2015). Modification of cellulose and its applications. Cellulose and Cellulose Derivatives.

[41] Sejie F.P., Matshwele J.T.P., Nareetsile F.M., Obuseng V.C. (2023). Nanocellulose surface modification reactions and their influence on the adsorption of heavy metal ions from water—A Review. Chem. Rev. Lett..

[42] Herbas-De la Cruz R.K., Choque-Quispe Y., Choque-Quispe D., Ligarda-Samanez C.A., Froehner S., Buleje-Campos D., Ramos-Pacheco B.S., Peralta-Guevara D.E., Pérez-Salcedo R., Yauris-Silvera C.R. (2023). Flocculant capacity of hydrocolloid extracted from high Andean algae (*Nostoc sphaericum*) in the treatment of artificial wastewater: An approach. Case Stud. Chem. Environ. Eng..

[43] Ligarda-Samanez C.A., Choque-Quispe D., Palomino-Rincón H., Ramos-Pacheco B.S., Moscoso-Moscoso E., Huamán-Carrión M.L., Peralta-Guevara D.E., Obregón-Yupanqui M.E., Aroni-Huamán J., Bravo-Franco E.Y. (2022). Modified Polymeric Biosorbents from *Rumex acetosella* for the Removal of Heavy Metals in Wastewater. Polymers.

[44] Oun A.A., Rhim J.-W. (2017). Characterization of carboxymethyl cellulose-based nanocomposite films reinforced with oxidized nanocellulose isolated using ammonium persulfate method. Carbohydr. Polym..

[45] Choque-Quispe D., Ligarda-Samanez C.A., Ramos-Pacheco B.S., Solano-Reynoso A.M., Quispe-Marcatoma J., Choque-Quispe Y., Peralta-Guevara D.E., Martínez-Huamán E.L., Correa-Cuba O., Masco-Arriola M.L. (2022). Formulation of Novel Composite (Activated Nanoclay/Hydrocolloid of *Nostoc sphaericum*) and Its Application in the Removal of Heavy Metals from Wastewater. Polymers.

[46] Singha A.S., Guleria A. (2014). Chemical modification of cellulosic biopolymer and its use in removal of heavy metal ions from wastewater. Int. J. Biol. Macromol..

[47] Andrade C. (2023). Understanding Statistical Noise in Research: 3. Noise in Regression Analysis. Indian J. Psychol. Med..

[48] Chen Q., Qi J. (2023). How much should we trust R^2^ and adjusted R^2^: Evidence from regressions in top economics journals and Monte Carlo simulations. J. Appl. Econ..

[49] Haroon H., Gardazi S.M.H., Butt T.A., Pervez A., Mahmood Q., Bilal M. (2017). Novel lignocellulosic wastes for comparative adsorption of Cr (VI): Equilibrium kinetics and thermodynamic studies. Pol. J. Chem. Technol..

[50] Choque-Quispe D., Ligarda-Samanez C.A., Choque-Quispe Y., Solano-Reynoso A.M., Ramos-Pacheco B.S., Zamalloa-Puma M.M., Álvarez-López G.J., Zamalloa-Puma A., Choque-Quispe K., Alzamora-Flores H. (2023). Multimetal removal in aqueous medium using a potato starch/nopal mucilage copolymer: A study of adsorption kinetics and isotherms. Results Eng..

[51] Simonin J.-P. (2016). On the comparison of pseudo-first order and pseudo-second order rate laws in the modeling of adsorption kinetics. Chem. Eng. J..

[52] Tseng R.-L., Wu F.-C., Juang R.-S. (2010). Characteristics and applications of the Lagergren’s first-order equation for adsorption kinetics. J. Taiwan Inst. Chem. Eng..

[53] Kostoglou M., Karapantsios T.D. (2022). Why Is the Linearized Form of Pseudo-Second Order Adsorption Kinetic Model So Successful in Fitting Batch Adsorption Experimental Data?. Colloids Interfaces.

[54] Ho Y.-S., McKay G. (1999). Pseudo-second order model for sorption processes. Process Biochem..

[55] Ho Y.S., McKay G. (1998). A comparison of chemisorption kinetic models applied to pollutant removal on various sorbents. Process Saf. Environ. Prot..

[56] Cheung C.W., Porter J.F., McKay G. (2001). Sorption kinetic analysis for the removal of cadmium ions from effluents using bone char. Water Res..

[57] Weber Jr W.J., Morris J.C. (1963). Kinetics of adsorption on carbon from solution. J. Sanit. Eng. Div..

[58] Oyelude E.O., Awudza J.A.M., Twumasi S.K. (2018). Removal of malachite green from aqueous solution using pulverized teak leaf litter: Equilibrium, kinetic and thermodynamic studies. Chem. Cent. J..

[59] Lima E.C., Sher F., Guleria A., Saeb M.R., Anastopoulos I., Tran H.N., Hosseini-Bandegharaei A. (2021). Is one performing the treatment data of adsorption kinetics correctly?. J. Environ. Chem. Eng..

[60] Tran H.N. (2023). Applying linear forms of pseudo-second-order kinetic model for feasibly identifying errors in the initial periods of time-dependent adsorption datasets. Water.

[61] Vareda J.P. (2023). On validity, physical meaning, mechanism insights and regression of adsorption kinetic models. J. Mol. Liq..

[62] Giacomni F., Menegazzo M.A.B., Silva M.G.d., Silva A.B.d., Barros M.A.S.D.d. (2017). Point of zero charge of protein fibers, an important characteristic for dyeing. Matéria.

[63] Kosmulski M. (2021). The pH dependent surface charging and points of zero charge. IX. Update. Adv. Colloid Interface Sci..

[64] Song J., He A., Jin Y., Cheng Q. (2013). Synthesis of amphoteric cellulose in aqueous NaOH–urea solution in one pot and its application in paper strength enhancement. RSC Adv..

[65] Zhu B., Fan T., Zhang D. (2008). Adsorption of copper ions from aqueous solution by citric acid modified soybean straw. J. Hazard. Mater..

[66] Largitte L., Pasquier R. (2016). A review of the kinetics adsorption models and their application to the adsorption of lead by an activated carbon. Chem. Eng. Res. Des..

[67] Anikushin B.M., Lagutin P.G., Kanbetova A.M., Novikov A.A., Vinokurov V.A. (2022). Zeta potential of nanosized particles of cellulose as a function of pH. Chem. Technol. Fuels Oils.

[68] Yadav S., Yadav A., Bagotia N., Sharma A.K., Kumar S. (2021). Adsorptive potential of modified plant-based adsorbents for sequestration of dyes and heavy metals from wastewater—A review. J. Water Process Eng..

[69] Leyva-Ramos R., Landin-Rodriguez L.E., Leyva-Ramos S., Medellin-Castillo N.A. (2012). Modification of corncob with citric acid to enhance its capacity for adsorbing cadmium (II) from water solution. Chem. Eng. J..

[70] Liu G., Liao L., Dai Z., Qi Q., Wu J., Ma L.Q., Tang C., Xu J. (2020). Organic adsorbents modified with citric acid and Fe_3_O_4_ enhance the removal of Cd and Pb in contaminated solutions. Chem. Eng. J..

[71] Li W., Ju B., Zhang S. (2019). A green l-cysteine modified cellulose nanocrystals biosorbent for adsorption of mercury ions from aqueous solutions. RSC Adv..

[72] Kamble S., Agrawal S., Cherumukkil S., Sharma V., Jasra R.V., Munshi P. (2022). Revisiting zeta potential, the key feature of interfacial phenomena, with applications and recent advancements. ChemistrySelect.

[73] Demirbolat M., Değim Z., Değim İ.T. (2021). Zeta potential determination of targeted nanoparticles. Drug Delivery with Targeted Nanoparticles.

[74] Valverde A., Cabrera-Codony A., Calvo-Schwarzwalder M., Myers T.G. (2024). Investigating the impact of adsorbent particle size on column adsorption kinetics through a mathematical model. Int. J. Heat Mass Transf..

[75] Zafari R., Fauteux-Lefebvre C. (2023). Surface modification investigation of nanocrystalline cellulose with combined functional groups for sulfur dioxide capture. Adsorption.

[76] Phaenark C., Jantrasakul T., Paejaroen P., Chunchob S., Sawangproh W. (2023). Sugarcane bagasse and corn stalk biomass as a potential sorbent for the removal of Pb (II) and Cd (II) from aqueous solutions. Trends Sci..

[77] Skoglund S., Hedberg J., Yunda E., Godymchuk A., Blomberg E., Odnevall Wallinder I. (2017). Difficulties and flaws in performing accurate determinations of zeta potentials of metal nanoparticles in complex solutions—Four case studies. PLoS ONE.

[78] Ravikanth P.V., Sudhakar B., Ramanmurhty K.V. (2017). Association of Particle Size and Zeta Potential—A Case Study with Propafol Injectable Lipid Emulsion. Lat. Am. J. Pharm..

[79] Aslam A.A., Hassan S.U., Saeed M.H., Kokab O., Ali Z., Nazir M.S., Siddiqi W., Aslam A.A. (2023). Cellulose-based adsorbent materials for water remediation: Harnessing their potential in heavy metal and dye removal. J. Clean. Prod..

[80] Johnson I., Kumar M. (2022). Algal-based biomaterials for environmental remediation of heavy metals. Biomass, Biofuels, and Biochemicals.

[81] Afolabi F.O., Musonge P., Bakare B.F. (2021). Evaluation of Lead (II) Removal from Wastewater Using Banana Peels: Optimization Study. Pol. J. Environ. Stud..

[82] Kang L., Liu S.-F., Yi D.-W., Wang K., Du H.-L., Huang H.-Q., Chen P. (2024). Renewable conversion of coal gangue to 13-X molecular sieve for Cd^2+^-containing wastewater adsorption performance. Rare Met..

[83] Zhu T., Zhang Y., Chen Y., Liu J.-L., Song X.-L. (2022). Synthesis of novel hydrated ferric oxide biochar nanohybrids for efficient arsenic removal from wastewater. Rare Met..

[84] Egbosiuba T.C., Egwunyenga M.C., Tijani J.O., Mustapha S., Abdulkareem A.S., Kovo A.S., Krikstolaityte V., Veksha A., Wagner M., Lisak G. (2022). Activated multi-walled carbon nanotubes decorated with zero valent nickel nanoparticles for arsenic, cadmium and lead adsorption from wastewater in a batch and continuous flow modes. J. Hazard. Mater..

[85] Yi Z.-J., Yao J., Kuang Y.-F., Chen H.-L., Wang F., Yuan Z.-M. (2015). Removal of Pb (II) by adsorption onto Chinese walnut shell activated carbon. Water Sci. Technol..

[86] Hasan R., Setiabudi H.D. (2019). Removal of Pb (II) from aqueous solution using KCC-1: Optimization by response surface methodology (RSM). J. King Saud Univ. Sci..

[87] Wu S., Jiang H., Lu J. (2023). Adsorptive performance and mechanism exploration of l-lysine functionalized celluloses for enhanced removal of Pb (II) from aqueous medium. Int. J. Biol. Macromol..

[88] Abdullah M., Abdullah L.C., Choong T.S.Y., Jamil S.N.A.M., Majid R.A., Adeyi A.A., Khalid A.K. (2022). Simultaneous adsorption of heavy metal ions (Cu^2+^ and Fe^2+^) from binary solutions by microcrystalline cellulose (MCC): Initial concentration effect, pH and kinetics studies. AIP Conf. Proc..

[89] Kaur M., Pal J. (2023). Evaluation of efficiency of wheat straw nanocellulose as nanoadsorbent for the removal of divalent copper, lead and zinc from aqueous solution. Carbohydr. Polym. Technol. Appl..

[90] Rahman A., Yoshida K., Islam M.M., Kobayashi G. (2023). Investigation of efficient adsorption of toxic heavy metals (chromium, lead, cadmium) from aquatic environment using orange peel cellulose as adsorbent. Sustainability.

[91] Issa N.B., Rajaković-Ognjanović V.N., Marinković A.D., Rajaković L.V. (2011). Separation and determination of arsenic species in water by selective exchange and hybrid resins. Anal. Chim. Acta.

[92] Takeda N., Fukushi K., Okuyama A., Takahashi Y. (2024). Solid–liquid partitioning and speciation of Pb (II) and Cd (II) on goethite under high pH conditions, as examined by subnanomolar heavy metal analysis, X-ray absorption spectroscopy, and surface complexation modeling. Chemosphere.

[93] Bayuo J., Rwiza M.J., Sillanpää M., Mtei K.M. (2023). Removal of heavy metals from binary and multicomponent adsorption systems using various adsorbents-a systematic review. RSC Adv..

[94] Lin G., Zeng B., Li J., Wang Z., Wang S., Hu T., Zhang L. (2023). A systematic review of metal organic frameworks materials for heavy metal removal: Synthesis, applications and mechanism. Chem. Eng. J..

[95] Xanthopoulou M., Giliopoulos D., Tzollas N., Triantafyllidis K.S., Kostoglou M., Katsoyiannis I.A. (2021). Phosphate removal using polyethylenimine functionalized silica-based materials. Sustainability.

[96] Wang C., Huang K., Mao L., Liang X., Wang Z. (2024). Incorporation of La/UiO66-NH_2_ into cellulose fiber for efficient and selective phosphate adsorption. J. Environ. Chem. Eng..

[97] Javanbakht V., Alavi S.A., Zilouei H. (2014). Mechanisms of heavy metal removal using microorganisms as biosorbent. Water Sci. Technol..

[98] Eloussaief M., Hamza W., Kallel N., Benzina M. (2013). Wastewaters decontamination: Mechanisms of PB (II), ZN (II), and CD (II) competitive adsorption on tunisian smectite in single and multi-solute systems. Environ. Prog. Sustain. Energy.

[99] Zheng Y., Ye H., Zhao G., Rao H., Liu H., Liu F., Wen N., Sun K. (2021). Competitive adsorption and correlative mechanism of heavy metal ions using ploy (cellulose/humic acid/acrylic acid) in multi-element aqueous medium. Polym. Bull..

[100] Pham H.G., Nguyen T.T.S., Pham T.T., Nguyen M.K., Vander Bruggen B., Nguyen T.T.M. (2023). Anionic competition on arsenate removal by modified granular ferric hydroxide adsorbent. Vietnam J. Sci. Technol. Eng..

[101] Xue S., Hu Y., Wan K., Miao Z. (2024). Exploring Humic Acid as an Efficient and Selective Adsorbent for Lead Removal in Multi-Metal Coexistence Systems: A Review. Separations.

[102] Wang Z., Lu Q., Liu C., Tian H., Wang J., Xie L., Liu Q., Zeng H. (2024). Nanoscale Insights into the Interaction Mechanism Underlying the Adsorption and Retention of Heavy Metal Ions by Humic Acid. Environ. Sci. Technol..

[103] Yang X., Han F., Xu C., Jiang S., Huang L., Liu L., Xia Z. (2017). Effects of preparation methods on the morphology and properties of nanocellulose (NC) extracted from corn husk. Ind. Crops Prod..

[104] Villabona-Ortíz Á., Tejada-Tovar C., Gonzalez-Delgado Á.D. (2021). Adsorption of Cd^2+^ Ions from Aqueous Solution Using Biomasses of *Theobroma cacao*, *Zea mays*, *Manihot esculenta*, *Dioscorea rotundata* and *Elaeis guineensis*. Appl. Sci..

[105] Miretzky P., Cirelli A.F. (2010). Cr (VI) and Cr (III) removal from aqueous solution by raw and modified lignocellulosic materials: A review. J. Hazard. Mater..

[106] Tejada-Tovar C., Herrera-Barros A., Villabona-Ortíz A. (2020). Assessment of chemically modified lignocellulose waste for the adsorption of Cr (VI). Fac. Ing..

[107] Garg R., Garg R., Sillanpää M., Alimuddin, Khan M.A., Mubarak N.M., Tan Y.H. (2023). Rapid adsorptive removal of chromium from wastewater using walnut-derived biosorbents. Sci. Rep..

[108] Chen M., Wang X., Zhang H. (2021). Comparative research on selective adsorption of Pb(II) by biosorbents prepared by two kinds of modifying waste biomass: Highly-efficient performance, application and mechanism. J. Environ. Manag..

[109] Herrera-Barros A., Tejada-Tovar C., Villabona-Ortíz A., Gonzalez-Delgado A.D., Alvarez-Calderon J. (2018). Adsorption of nickel and cadmium by corn cob biomass chemically modified with alumina nanoparticles. Indian J. Sci. Technol..

[110] Yang J., Hou B., Wang J., Tian B., Bi J., Wang N., Li X., Huang X. (2019). Nanomaterials for the removal of heavy metals from wastewater. Nanomaterials.

[111] Hu T., Hu X., Tang C., Liu D. (2022). Adsorbent grafted on cellulose by in situ synthesis of EDTA-like groups and its properties of metal ion adsorption from aqueous solution. Cellulose.

[112] Tejada-Tovar C., Villabona-Ortiz Á., Garcés-Jaraba L. (2015). Adsorption of heavy metals in waste water using biological materials. TecnoLógicas.

[113] Mangood A.H., El-Saied F.A., El-shinawy F.H., Abo-Elenan S.A. (2024). Metals Removal from Contaminated Aqueous Medium by Using Modified Cellulose-N (4)-Antipyrinyl Thiosemicarbazide. Polycycl. Aromat. Compd..

[114] Schick A., Zhu Y., Du X. (2018). Estimation of the error distribution in a varying coefficient regression model. J. Nonparametric Stat..

[115] Bangaraiah P., Sarathbabu B. (2019). Optimization of process parameters in removal of lead from aqueous solution through response surface methodology. Chem. Eng. Commun..

[116] Bahrami M., Amiri M.J., Bagheri F. (2019). Optimization of the lead removal from aqueous solution using two starch based adsorbents: Design of experiments using response surface methodology (RSM). J. Environ. Chem. Eng..

[117] Bayuo J., Pelig-Ba K.B., Abukari M.A. (2019). Optimization of adsorption parameters for effective removal of lead (II) from aqueous solution. Phys. Chem. Indian J..

[118] Asif Z., Chen Z. (2017). Removal of arsenic from drinking water using rice husk. Appl. Water Sci..

[119] Bui T.H., Pham V.S., Thanh-Nho N., Trieu Q.A. (2021). Removal of arsenic from water using a composite of iron–manganese oxide incorporated active rice husk silica. CLEAN Soil Air Water.

[120] Iqbala D.N., Yousafa H., Masoodb N., Iqbala M., Nazira A. (2020). Factors affecting the efficiency of rye husk as a potential biosorbent for the removal of metallic pollutants from aqueous solutions. Mar. Life.

[121] Babazad Z., Kaveh F., Ebadi M., Mehrabian R.Z., Juibari M.H. (2021). Efficient removal of lead and arsenic using macromolecule-carbonized rice husks. Heliyon.

[122] Zhou D., Zhang L., Zhou J., Guo S. (2004). Cellulose/chitin beads for adsorption of heavy metals in aqueous solution. Water Res..

[123] Matouq M., Jildeh N., Qtaishat M., Hindiyeh M., Al Syouf M.Q. (2015). The adsorption kinetics and modeling for heavy metals removal from wastewater by Moringa pods. J. Environ. Chem. Eng..

[124] Cavus S., Gurdag G. (2009). Noncompetitive removal of heavy metal ions from aqueous solutions by poly [2-(acrylamido)-2-methyl-1-propanesulfonic acid-co-itaconic acid] hydrogel. Ind. Eng. Chem. Res..

[125] Zhang J. (2019). Physical insights into kinetic models of adsorption. Sep. Purif. Technol..

[126] Jiménez B.J., Boscov M.E.G. Adsortion kinetics of cadmium, lead, and nickel in vermiculite. Proceedings of the Geo-Chicago 2016.

[127] Islam M.S., Rahaman M.S., Barbeau B. (2023). Removal of Pb (II), Zn (II), Cu (II), and As (III) ions from water using kraft pulp-based carboxymethylated cellulose in a fixed-bed column adsorption process. J. Environ. Chem. Eng..

[128] Liu J., Xie T.-H., Deng C., Du K.-F., Zhang N., Yu J.-J., Zou Y.-L., Zhang Y.-K. (2014). Welan gum-modified cellulose bead as an effective adsorbent of heavy metal ions (Pb^2+^, Cu^2+^, and Cd^2+^) in aqueous solution. Sep. Sci. Technol..

[129] Efimova N.V., Krasnopyorova A.P., Yuhno G.D., Scheglovskaya A.A. (2017). Sorption of heavy metals by natural biopolymers. Adsorpt. Sci. Technol..

[130] Šuránek M., Melichová Z., Kureková V., Kljajević L., Nenadović S. (2021). Removal of nickel from aqueous solutions by natural bentonites from slovakia. Materials.

[131] Kozlov V.A., Nikiforova T.E., Islyaikin M., Koifman O.I. (2017). The mechanism of d-metal ion sorption from aqueous media and chelating sites structures of modified heterocyclic biopolymers. Can. J. Chem..

[132] Vázquez-Guerrero A., Cortés-Martínez R., Alfaro-Cuevas-Villanueva R., Rivera-Muñoz E.M., Huirache-Acuña R. (2021). Cd (II) and Pb (II) adsorption using a composite obtained from moringa oleifera lam. Cellulose nanofibrils impregnated with iron nanoparticles. Water.

[133] He Z., Song H., Cui Y., Zhu W., Du K., Yao S. (2014). Porous spherical cellulose carrier modified with polyethyleneimine and its adsorption for Cr (III) and Fe (III) from aqueous solutions. Chin. J. Chem. Eng..

[134] Barquilha C.E.R., Cossich E.S., Tavares C.R.G., Silva E.A. (2019). Biosorption of nickel (II) and copper (II) ions by *Sargassum* sp. in nature and alginate extraction products. Bioresour. Technol. Rep..

[135] Wang N., Qiu Y., Xiao T., Wang J., Chen Y., Xu X., Kang Z., Fan L., Yu H. (2019). Comparative studies on Pb (II) biosorption with three spongy microbe-based biosorbents: High performance, selectivity and application. J. Hazard. Mater..

[136] He H., Lee J., Jiang Z., He Q., Dinic J., Chen W., Narayanan S., Lin X.-M. (2023). Kinetics of Shear-Induced Structural Ordering in Dense Colloids. J. Phys. Chem. B.

[137] Hajeeth T., Sudha P.N., Vijayalakshmi K., Gomathi T. (2014). Sorption studies on Cr (VI) removal from aqueous solution using cellulose grafted with acrylonitrile monomer. Int. J. Biol. Macromol..

[138] Khamizov R.K. (2020). A pseudo-second order kinetic equation for sorption processes. Russ. J. Phys. Chem. A.

[139] Tighadouini S., Radi S., Roby O., Hammoudan I., Saddik R., Garcia Y., Almarhoon Z.M., Mabkhot Y.N. (2022). Kinetics, thermodynamics, equilibrium, surface modelling, and atomic absorption analysis of selective Cu(ii) removal from aqueous solutions and rivers water using silica-2-(pyridin-2-ylmethoxy)ethan-1-ol hybrid material. RSC Adv..

[140] Ma M.-Y., Ke X., Liu Y.-C., Ha Z.-H., Wang T., Li J., Zhang F., Zhang T.C. (2023). A novel electrolytic-manganese-residues-and-serpentine-based composite (S-EMR) for enhanced Cd (II) and Pb (II) adsorption in aquatic environment. Rare Met..

[141] Wang J., Guo X. (2022). Rethinking of the intraparticle diffusion adsorption kinetics model: Interpretation, solving methods and applications. Chemosphere.

[142] Seidi F., Reza Saeb M., Huang Y., Akbari A., Xiao H. (2021). Thiomers of chitosan and cellulose: Effective biosorbents for detection, removal and recovery of metal ions from aqueous medium. Chem. Rec..

[143] Marrane S.E., Dânoun K., Essamlali Y., Aboulhrouz S., Sair S., Amadine O., Jioui I., Rhihil A., Zahouily M. (2023). Fixed-bed adsorption of Pb (ii) and Cu (ii) from multi-metal aqueous systems onto cellulose-g-hydroxyapatite granules: Optimization using response surface methodology. RSC Adv..

